# 2199 Critical and creative thinking course: Fundamental for a junior researcher

**DOI:** 10.1017/cts.2018.200

**Published:** 2018-11-21

**Authors:** Lourdes E. Soto de Laurido, Marie K. Norman, Doris Rubio

**Affiliations:** University of Puerto Rico-Medical Sciences Campus

## Abstract

OBJECTIVES/SPECIFIC AIMS: Explain the difference between creative and critical thinking. Practice and enhance the critical thinking skills. Display innovative thinking through creative solutions and insights. Critically evaluate evidence in research. Think imaginatively, actively seeking out new points of view. METHODS/STUDY POPULATION: Offer an online course in Critical and Creative Thinking to junior researchers to improve their capacity to think and transforms their ideas in research questions and aims that bring new option to the field of clinical and translational research. Evaluate their improvement through evaluation forms and exercises that show their process to think imaginatively. RESULTS/ANTICIPATED RESULTS: The Scholars will understood the importance of critical and creative thinking in their careers, believed they could apply the insights and knowledge from the course in their grant and paper writing, recognized that they don’t always consider if they are being critical or creative in their thinking and actions. DISCUSSION/SIGNIFICANCE OF IMPACT: The course helped the participants to improve their capacity to think and saw a need to develop a more systematic thought processes in their life and work. The junior research will understand the difference between opinion, reasoned, judgment and fact and they will be able to judge the credibility of an information source using criteria such as authorship, currency and potential bias that can improve their grant submission and scientific writing skills.

